# National Health Insurance Notes

**Published:** 1924-05

**Authors:** 


### NATIONAL HEALTH INSURANCE NOTES.

### OUT OF THE HAT.

The facility with which the money for the doctors has been found without either raiding the funds of Approved Societies or milking the Exchequer cow would be amusing were it not that all the clamour and tumult on the subject for many months must have done the Insurance medical service a great deal of harm. By the Minister's scheme of taking seven-ninths of twopence from this source, and seven-ninths of one-and-something-farthing from that, and a bit from the other, faces have been saved, pledges observed to the letter, fights on the floor of the House avoided. The Societies can now begin to talk freely, as some of them are already doing, of the millions with which they will be able to play when the results of the current valuation become known. Mr. Wheatley is depicted in *Punch* as producing the money out of a hat. We, whose eyes have been washed, can see a little further. We recognise the hat. It is the one through which the Approved Societies have been talking for some time past.

### THE NEW CERTIFICATION RULES.

The little initial mistake of printing on the form to be handed to a patient a skeleton announcement of his forthcoming death having been surmounted (it followed rather closely on the scandal of the margarine advertisement) the doctors and the Societies are now getting into their stride with the revised certification arrangements. These embody many valuable improvements. The new forms of voluntary certificate and death certificate are advantageous both to Societies and doctors, as also is the inclusion of all other types of certificate on two forms. The doctor is relieved of the obligation of giving any certificate to an insured person who is not actually being treated by him—that is, one who is receiving treatment at an institution or from some unqualified person. The Societies welcome the rule that all certificates, in addition to bearing the doctor's signature, are to be clearly stamped with his name and address, and also the new provision that the doctor, when he is aware that a patient's Society is giving extra benefits in regard to which a medical recommendation is useful, shall give such a recommendation on the form of certificate.

### CO-OPERATION.

If doctors fully carry out their obligations under the certification rules with a jealous regard for their own reputations, and the Societies will rigorously refrain, as they have once again been asked to do, from requesting doctors to give certificates irregularly, all will be well. The Societies have also been requested not to require certificates on particular days to fit in with their pay-days; at the same time, a doctor can often do this without prejudicing the patient's treatment or giving himself any appreciable extra trouble. Co-operation in this and other matters, due to a mutual understanding of the other man's difficulties, can do wonders in the smooth working of the certification machinery.

### FINED A THOUSAND POUNDS.

With the technicalities of the case in Lancashire in which a thousand pounds was withheld from the payment to two doctors in partnership for offences under the regulations we are not concerned. The penalty itself is another matter. When the late Minister imposed a penalty of this amount, he may possibly have considered, as a matter of arithmetic, that it was a far smaller penalty than the capitalised loss which removal from the panel would involve. There is, however, a psychological aspect of the question which cannot be ignored. The one penalty is shocking, in the literal sense of the word; the other is not. We express no opinion on the merits of the case in question, but there is a general feeling that, where so large an amount comes into play, this type of punishment does not "fit the crime."

### PROTECTING THE DOCTOR.

A patient's name has been removed from a doctor's list, and the patient is told by the doctor, on being summoned to attend him, that he will do so if the patient undertakes to pay his fee; this the patient, being convinced that his name has been wrongly taken off the doctor's list, refuses to do. Is the doctor justified in refusing to attend? It seems not unreasonable, and the Minister, in a recent decision on an appeal by a doctor from the decision of an Insurance Committee, has so determined. Mistakes are often made in the lists of insured persons furnished to the doctors; they are not always avoidable. The doctor in such cases is expressly required to attend, but (the regulation proceeds) he may demand a fee. This right to demand a fee, which, of course, is afterwards to be refunded if the patient is found to be an insured person, is held to limit the doctor's obligation.

---

### MORE TEACHING IN HOSPITALS.

The editorial columns of the *Long Island Medical Journal* call attention to two important movements in American medicine. With remedies for the deplorable traffic in medical degrees, we are, fortunately, not concerned. But the question of how to make post-graduate study more accessible to the average practitioner is especially important on this side of the Atlantic. The situation in the United States appears to be that the great city hospitals are subjected to so heavy and increasing a demand for teaching facilities that the time has come when smaller institutions must take up their part of the burden. Whether they become medical school hospitals or not, they must open their doors to those who seek instruction. To go even further, it is suggested that they should all participate in some way in the general educational programme of the community which they serve. On these lines active organisation is proceeding in many American States, and although it is too soon to give details of their arrangements, it is at least justifiable again to call our own authorities' attention to the various schemes now under discussion in America.

